# Adapting bioinformatics curricula for big data

**DOI:** 10.1093/bib/bbv018

**Published:** 2015-03-30

**Authors:** Anna C. Greene, Kristine A. Giffin, Casey S. Greene, Jason H. Moore

**Keywords:** big data, bioinformatics, data science, education

## Abstract

Modern technologies are capable of generating enormous amounts of data that measure complex biological systems. Computational biologists and bioinformatics scientists are increasingly being asked to use these data to reveal key systems-level properties. We review the extent to which curricula are changing in the era of big data. We identify key competencies that scientists dealing with big data are expected to possess across fields, and we use this information to propose courses to meet these growing needs. While bioinformatics programs have traditionally trained students in data-intensive science, we identify areas of particular biological, computational and statistical emphasis important for this era that can be incorporated into existing curricula. For each area, we propose a course structured around these topics, which can be adapted in whole or in parts into existing curricula. In summary, specific challenges associated with big data provide an important opportunity to update existing curricula, but we do not foresee a wholesale redesign of bioinformatics training programs.

## Big data challenges

The modern quantitative scientist is awash in a data deluge. The amount of data being generated far outweighs that being thoroughly analyzed. For example, Wal-mart stores process more than 1 million customer transactions per hour, and users upload >100 h of video content per minute on YouTube [1, 2]. It is clear that some data are ‘big’ and that such big data come in many forms and are of great interest to a variety of groups, from biologists to law enforcement, social services, private companies and homeland security [3]. There is no consensus on what constitutes big data [4, 5]. In general, the concept encompasses collections that are too large to manage and analyze using traditional approaches. Under this model, what specifically constitutes big data is a field-specific moving target that grows as research advances.

The data that meet this definition in biology and medicine are generated from numerous sources, including laboratory experiments, medical records and insurance/claims data [6] and are accessible via online databases such as the ArrayExpress repository [7], the eMERGE network [8] and the SEER-Medicare database, respectively [9]. Biomedical big data are emerging from the combination of small data sources as well. For example, as scientists share their laboratory experiments with others in ArrayExpress, this creates a resource containing >54 000 genome-wide experiments measuring >1.6 million conditions [7]. These aggregate big data are inherently economical to use, as the cost of data generation is shared over many labs, and computational methods have been developed to use these aggregate data [10–14].

Biomedical big data provide the opportunity to develop data-driven predictions that complement knowledge-based hypothesis generation. Because these data represent multi-investigator and multi-institution resources, the systems being measured are diverse and discoveries are expected to be more likely to generalize [15–17]. Big data present new opportunities, as well as new challenges. Adapting bioinformatics curricula to address these challenges will require us to develop curricula that provide the skills to harness big data and the skepticism to critically evaluate findings.

The challenges raised by big data that our curricula should prepare students to address include data unification [18], computational and storage limitations [6, 18, 19], multiple hypothesis testing [6] and bias and confounding in the data [6]. Data unification encompasses the challenges of both data wrangling, i.e. obtaining the necessary data in the appropriate format, as well as the normalization necessary to make them comparable across sources. Computational and storage limitations refer to the difficulties and costs associated with keeping data, moving data and analyzing data. Multiple hypothesis testing refers to the challenge of statistically addressing the likelihood of finding spurious associations in large data sets. Bias and confounding in the data refer to challenges related to which experiments have been performed or which processes are most frequently assayed. The field is moving rapidly, and the challenges and the solutions to them are not static. Bioinformatics trainees in the big data era will need to be able to understand the current computing environment (processor, storage, memory and network costs) and how to, within that environment, most effectively analyze and gain insights from large-scale data. We propose updates to curricula to address these key factors.

## Investments in big data training and research

Significant resources are being allocated for training scientists in the analysis of large-scale data. Recent US governmental investments including the Big Data Initiative [20] and the NIH Big Data to Knowledge (BD2K) initiative [21] focus on research and training related to big data challenges. The Big Data Initiative specifically aims to ‘greatly improve the tools and techniques needed to access, organize, and glean discoveries from huge volumes of digital data’ [20]. The 200 million dollar investment is divided across six federal departments and agencies and will fund both student training initiatives as well as research programs. One component of the BD2K initiative is to train researchers who can harness the power of big data [21]. The University of Washington recently received a 2.8 million dollar National Science Foundation Integrative Graduate Education and Research Traineeship (IGERT) grant entitled ‘Big Data U’ that aims to train graduate students to use big data in many different fields of research [22]. In the UK, the Medical Research Council has established a 90 million pound initiative to support big data challenges [23]. IBM has partnered with the Ministry of Education and 100 universities in China to promote big data and analytics training programs through a 100 million dollar investment [24].

Private foundations have also invested heavily in training students to transform big data into new discoveries. The Gordon and Betty Moore Foundation and Alfred P. Sloan Foundation are supporting New York University (NYU), The University of California, Berkeley, and the University of Washington with a cross-institutional 5 year 37.8 million dollar initiative to advance data-driven discovery that will allow ‘university researchers to harness the full potential of the data-rich world that characterizes all fields of science and discovery’ [25]. A primary component of this award is to empower education in data science [25]. The Li Ka Shing Foundation has given funds to promote big data research at Oxford University and Stanford University [26, 27].

Significant resources are also being allocated for the analysis of big data, and trainees of bioinformatics programs that update their curricula for big data would be ideal competitors for these grants, such as the BD2K and Big Data initiatives. The Gordon and Betty Moore Foundation has directly funded academic researchers through their 5 year data-driven discovery grants as well [28].

Through these large governmental and private investments, graduate students will have greater access to big data resources and opportunities to learn from experts in the field, which will aid in addressing some of the challenges that big data present. These investments also ensure that students will be properly trained in extracting rich information found in big data, and they will fill a pipeline of well-trained scientists capable of working with big data.

## Changes in bioinformatics training programs and the arrival of new data science programs over the past 12–18 months

Bioinformatics curricula at the undergraduate [29–34] and graduate level [29–33, 35–38] have been reviewed previously, and here we focus on contributions to the field in graduate education in between 2012 and 2014. Margolis *et al*. [21] state that constituencies in the ‘biomedical big data ecosystem include data providers and users (eg, biomedical researchers, clinicians, and citizens), data scientists, funders, publishers, and libraries’. To create and maintain this ecosystem, bioinformatics education is a critical component. We must train scientists who are able to work effectively with biomedical big data. Bioinformatics programs at every degree level exist globally; however, the extent to which these programs have updated their curricula to accommodate the rapidly changing environment of bioinformatics training is unknown. The evolving training landscape is currently driven by the creation of data science centers and departments that offer degree programs that mirror the computational science and statistical coursework offered by bioinformatics programs. The primary difference between many bioinformatics curricula and these new data science programs is the specific focus on biological problems in bioinformatics versus a wider array of topics found in data science, from business analytics to data security. Data science programs may also delve more deeply into computational, mathematical and statistical sciences (e.g. required courses in statistical inference, data visualization, machine learning and data management). An exciting aspect of data science programs is that they are extremely transdisciplinary, perhaps more so than traditional bioinformatics programs, as the data being analyzed span many disparate fields.

For example, The Center for Data Science at NYU offers a masters degree in data science and also launched the Moore-Sloan Data Science Environment which allows scientists in fields such as biology or astrophysics to collaborate across the mathematical and computational sciences. Indiana University, Columbia University, Carnegie Mellon University, NYU and Worcester Polytechnic Institute are just a few of the universities that have rolled out data science graduate programs; there are many additional schools that offer certification in data science. These collaborative data science programs are likely to positively impact bioinformatics programs, as there should be cross-talk between them through faculty interactions and cross-listed courses. This synergistic environment extends beyond the confines of single institutions through collaborative big data grants such as the Gordon and Betty Moore Foundation and Alfred P. Sloan Foundation initiative between NYU, UC-Berkeley and the University of Washington, which focus on ‘championing education and training in data science at all levels’ [25].

Many of these programs are addressing the challenge of training domain experts in hard analytical skills while simultaneously training computational researchers in the necessary domain knowledge. While there are diverse opinions about how to update bioinformatics curricula to stay relevant in the era of big data, one must also remember that enacting those updates and training at the interface of multiple fields is inherently difficult. Graduate training in bioinformatics is difficult because it is a rapidly evolving field, and many new graduate students come from one field of strength (i.e. computer science or biology), rather than an interdisciplinary one. Abeln *et al*. [39] address this with a fresh perspective on interdisciplinary education. Their novel MSc program housed between VU University Amsterdam and the University of Amsterdam allows students to choose a track in bioinformatics or systems biology [39]. At the start of their program, the required courses are taken alongside ‘conversion courses’, which aim to bring students up to speed in two of three areas, wherever they may be lacking expertise (molecular biology, mathematics and programming). Throughout their program, the classes have interdisciplinary projects that reinforce concepts across the disciplines. Below, we propose additions to current bioinformatics curricula, and to accommodate the growing body of knowledge that students need to learn in the era of big data, these additions may be difficult to fit into already challenging curricula. However, innovative approaches to interdisciplinary education, such as the one proposed by Abeln *et al*., are one way that programs may adapt their curricula to train students more broadly in the big data deluge.

## Additions to existing curricula

An important question to consider now is whether current bioinformatics training programs are adjusting their curricula to incorporate big data challenges. Individuals with the ability to analyze, organize and foster discoveries from immense volumes of data are valuable in today’s economy and will become more so in the future. According to a report filed by McKinsey and Company where they studied the economic impact of big data on different economic sectors, there will be a potential shortfall of 140 000–190 000 people for positions requiring ‘deep analytical skills’ by 2018 [4]. Many bioinformatics programs were established before big data became a prominent area of focus. These programs may need to refresh some aspects of their curriculum to stay competitive among other programs and to remain competitive for funding sources. While many aspects of data science curricula now represent important areas of bioinformatics, the modern bioinformatics researcher should have extensive knowledge of biology, which is likely to extend beyond that covered in existing data science curricula. Bioinformatics students in general benefit from an interdisciplinary training program that trains broadly in computer science, statistics, bioinformatics and biology. Bioinformatics curricula updates should address data unification [18], computational and storage limitations [6, 18, 19], multiple hypothesis testing [6] and bias and confounding in the data [6].

Bioinformatics curricula have generally focused on teaching students how to develop computationally efficient solutions to pressing biological challenges. We are now seeing a shift from process-improvement solutions (e.g. improved pipelines) to data-driven discovery solutions (e.g. improved algorithms). Below we include suggested courses on ‘The Flow of Biological Information’, ‘Statistical Challenges of Big Data’ and ‘Computational Challenges of Big Data’. These hypothetical courses highlight some of the most necessary biology, computational and statistical knowledge needed by the next generation of bioinformaticists for the era of big data and provide a framework for the redesign of program curricula. While we suggest these as courses that may be added to existing curricula, these skills may also be acquired through required coursework, electives, journal clubs and online resources. These suggested courses include topic areas that should be covered in a bioinformatics curriculum, but more work is needed to implement and assess the impact of training program updates. As noted in Magana *et al*. [40], while the bioinformatics community has broadly implemented curricula, little is known about the effectiveness of these curricula on learning objectives. As with any graduate curriculum, it should be tailored to the student’s strengths and deficiencies to enable the greatest chance for success.

To remain at the forefront of bioinformatics education, curricula should be updated on an annual or biennial basis. Course instructors should adjust curricula as necessary based on advancements in the field. They should also engage with current students in their classes to assess class activity and topic usefulness, obtain class evaluations during and after the class ends, as well as stay in contact with class/program alumni to discuss the impact of the class on their success after graduation [41]. Additionally, it is helpful in an interdisciplinary program like bioinformatics to have regular faculty meetings across the disciplines in the core curricula to communicate how the program curricula should evolve as a whole based on advancements in their respective fields.

## Suggested additional course: The Flow of Biological Information

While a broad overview of molecular biology is most thoroughly accomplished through a series of graduate courses in biology, it is not always feasible to incorporate a lengthy course series into existing curricula. Professor Russ Altman of Stanford University, in 1998, published five areas of competence for bioinformatics training: biology, computer science, statistics, ethics and core bioinformatics [35]. In a recent update [42], Professor Altman said, ‘For biomedicine, there is little doubt that the best data scientists will be those who understand the special features and challenges in biology or medicine, and thus make assumptions and approximations that are valid and not fatal’.

**Figure 1.**
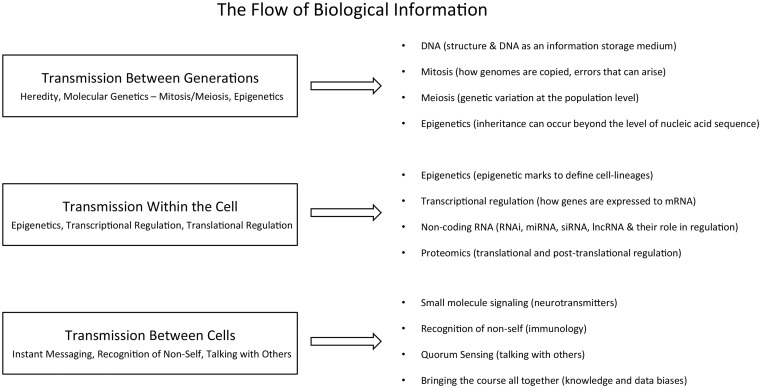
Proposed course on the flow of biological information.

The large-scale data being generated in biology measure genome sequences, gene expression, protein signaling and other areas of information flow through complex biological systems. We describe a blended molecular biology and genetics course entitled ‘The Flow of Biological Information’, which would serve as a primer on the areas of biology that are frequently encountered by bioinformatics students (Figure 1). This course is intended to provide students with an understanding of the measurements behind their data. Professor Lawrence Hunter from the University of Colorado Denver emphasized the importance of this knowledge when he said [42], ‘Insights into the idiosyncrasies of instruments such as mass spectrometers and hybridization arrays have led to dramatic improvements in informatics methods not available to those who treat data as a “given”’. In addition to developing an understanding of the underlying experimental platforms, this course will familiarize students with the data formats and skills necessary for data wrangling in this domain [43].

Each week covers an important type of biology information storage and processing. The conceptual lectures are paired with a lab in which the students analyze data relevant to the week’s lecture using recently developed methods. During lectures focused on each type of information, students will be exposed to the types of data that are commonly used to measure these systems. To effectively carry out these labs, students should have a strong programming background when coming into the course. A common theme throughout the course should be the data and knowledge biases associated with each type of information.

The course is organized around key types of information transfer, organized into three modules. The first module is Transmission Between Generations (heredity, molecular genetics—mitosis/meiosis, epigenetics) with lectures in DNA (structure and DNA as an information storage medium), mitosis (how genomes are copied, errors that can arise), meiosis (genetic variation at the population level) and epigenetics (inheritance can occur beyond the level of nucleic acid sequence). Example labs relating to these lecture topics include aligning reads to a genome (e.g. Bowtie [44]), identification of somatic variations in cancer (e.g. apply MuTect [45]), 1000 genomes/variant annotations (e.g. [46]) and bisulfite sequencing alignment (e.g. [47]). The second module covers Transmission Within the Cell [epigenetics, transcriptional regulation (mRNA, miRNA and siRNA) and translational regulation (protein, phosphorylation)] and has lectures on epigenetics (epigenetic marks as a means to define cell-lineages), regulation of transcription (transcription factor binding, motifs, ChIP-seq), steady-state mRNA levels (how genes are expressed to mRNA), noncoding RNA (RNAi, miRNA, siRNA, lncRNA and their role in regulation) and proteomics (translational and posttranslational regulation). The last module is Transmission Between Cells [instant messaging (small molecule signaling), recognition of nonself (immunology) and talking with others (quorum sensing)] and discusses small molecule signaling (neurotransmitters, etc), recognition of nonself and quorum sensing. The course is capped by a student-defined final project through which they integrate two or more data types covered within the course.

## Suggested additional course: Statistical Challenges of Big Data

For example, the uncertainty around results represents an important and fast-moving area. In an interview about the challenges of big data, Michael I. Jordon from the University of California, Berkeley, states, ‘We have to have error bars around all our predictions. That is something that’s missing in much of the current machine learning literature’ [48]. He likens this to bridge building: ‘If I have no principles, and I build thousands of bridges without any actual science, lots of them will fall down, and great disasters will occur’ [48]. He states that prediction error bars will take years of research, highlighting one reason why bioinformatics education in big data analysis is important and will require curricula to be updated as the field evolves.

Analyzing big data presents additional statistical challenges. In such data, even infrequent observations are expected to be frequent. As The Whitlams’ song Up Against the Wall notes, ‘She was one in a million, yeah; So there's five more, just in New South Wales’ [49]. In a data set with a trillion observations, we expect 1000 ‘one in a billion’ events. Experiments to evaluate results from the analysis of big data need to take this into account.

**Figure 2.**
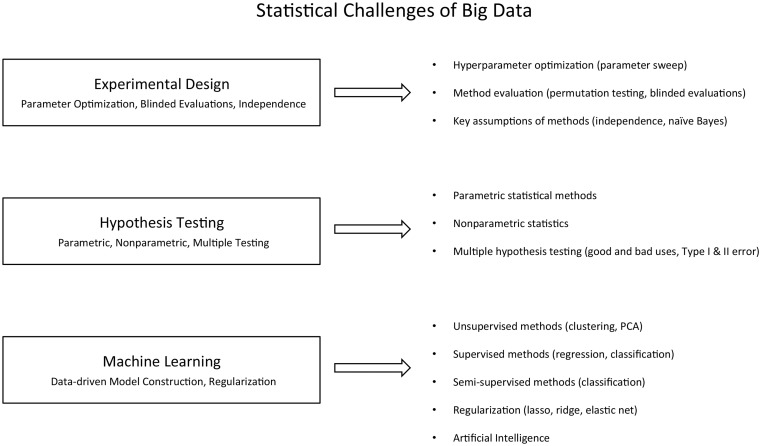
Proposed course on the statistical challenges of big data.

This course is organized around principles of experimental design, hypothesis testing and machine learning and is paired each week with an associated lab that reinforces the concepts learned during the week’s lectures (Figure 2). This course would include motivating examples from recent literature and would use data sets for data types covered in the transmission of information course. A strong emphasis would be placed on the critical evaluation of potential discoveries, with specific training toward recognizing knowledge or data biases that could lead to spurious discoveries.

The first key module is Experimental Design (parameter optimization, blinded evaluations, independence) and has lectures on hyperparameter optimization (parameter sweep), method evaluation (permutation testing, blinded evaluations) and key assumptions of methods (independence, naïve Bayes). Example labs for these lectures include a parameter sweep for an appropriate ‘c’ using support vector machines (SVM), evaluation to see if this SVM works better than random and using a naïve Bayes classifier to combine simulated data with nonindependence and/or random data (which is worse?). The second module is based on Hypothesis Testing (parametric, nonparametric, multiple testing) and features lecture topics on parametric statistical methods, nonparametric statistics and multiple hypothesis testing (good and bad uses, Type I and II error). The last module focuses on machine learning (data-driven model construction, regularization), with lectures on unsupervised methods (clustering, PCA), supervised methods (regression and classification), semi-supervised methods (classification), regularization (lasso, ridge, elastic net) and artificial intelligence. The conclusion of the course is focused on a student project to use all of the knowledge gained from the course to analyze a new data set, extract novel insights and analyze the validity of the results.

## Suggested additional course: Computational Challenges of Big Data

**Figure 3.**
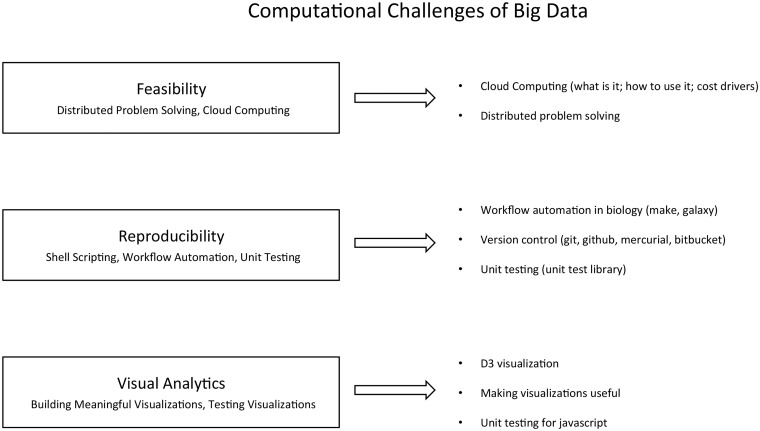
Proposed course on the computational challenges of big data.

Analyzing big data present new computational challenges. These data may need to be analyzed in a distributed manner, the analyses performed should be highly reproducible to allow for thorough evaluation of potential biases, and results need to be presented as effective visual summaries. Our proposed course touches on each of these areas (Figure 3).

To address the challenge of feasibility, we introduce students to cloud computing. Vasant Dhar, Professor and Director of the Center for Business Analytics at NYU’s Stern School of Business, recently highlighted cloud computing and distributed computing as necessary components of a data science education [50]. To address reproducibility, we introduce workflow automation, version control and unit testing [51–53]. For visualization, we use the modern javascript library, d3.js, to teach students to make high-content visualizations of complex data sets.

While each of these topics is large enough to be covered in an individual course, the emphasis of this course is on providing students with the groundwork necessary to identify the appropriate courses and focus areas for a given challenge. The laboratory components of this course would be conducted entirely using cloud computing resources (e.g. through Amazon’s EC2). This would provide students with efficient solutions for multiple types of data, expertise in managing a cloud computing system and an important background in conveying results to a broader audience.

The first module is Feasibility (distributed problem solving and cloud computing) with lectures in cloud computing (what is it, how to use it and cost drivers) and distributed problem-solving. Example labs include creating a cloud instance and performing an analysis, implementing a parameter sweep/embarrassingly parallel process and read mapping across many reads or building a model with many examples. The second module focuses on Reproducibility (shell scripting, workflow automation and unit testing) and contains lectures on workflow automation in biology (make, galaxy, etc), version control (git, github, mercurial and bitbucket) and unit testing (unit test library for a programming language of choice). The last module is Visual Analytics (building meaningful visualizations, testing visualizations), including topics on D3 visualization, making visualizations useful and unit testing for javascript. The course is capped by a project in which the students integrate what they have learned in this course with the biology and statistics components of the curriculum. The final project uses cloud computing to do an analysis that is infeasible on a single machine using an automated workflow stored in a version control system and to visualize the results in an informative way.

## Conclusion

We are living in a data revolution, where four dimensions define big data: volume, velocity, variety and veracity [54]. Because of the complexity of biomedical big data, there is a growing need to produce bioinformatics professionals that are capable of processing, analyzing and interpreting big data. While big data bring great promise, there are many associated challenges with it as well. There is a need to update bioinformatics curricula to address these challenges, which requires us to develop curricula that provide the appropriate skillset for analyzing big data along with the knowledge to extract and determine the validity of key findings. Curricula updates should address data unification [18], computational and storage limitations [6, 18, 19], multiple hypothesis testing [6] and bias and confounding in the data [6]. We have also proposed three courses in ‘The Flow of Biological Information’, ‘Statistical Challenges of Big Data’ and ‘Computational Challenges of Big Data’. These courses feature the most necessary biology, computational and statistical knowledge needed for students to graduate as well-informed bioinformaticians in the era of big data. The courses may be added to current bioinformatics curricula or may serve as a jumping off point for the inclusion of new lectures into courses already being taught. Additionally, many schools now offer data science programs. The coursework housed in these programs may facilitate educational updates to bioinformatics programs through the cross-listing of relevant courses and other scholarly activities. As Welch *et al*. [33] showed, companies are now looking for bioinformaticians who are able to work effectively with big data, and thus, we must augment bioinformatics curricula to appropriately prepare our students for the world after graduation.

**Key Points**

- The phrase ‘big data’ has emerged as a catchall for data and information that is so big that it is difficult to store, transport, manipulate, analyze and interpret.
- There is a growing need to produce bioinformatics professionals who are capable of processing, analyzing and interpreting big data.
- We propose updates to current bioinformatics curricula that include topics such as data unification, computational and storage limitations, multiple hypothesis testing and bias and confounding in the data.
- We have also proposed three courses in ‘The Flow of Biological Information’, ‘Statistical Challenges of Big Data’ and ‘Computational Challenges of Big Data’.

## Funding

National Institutes of Health [grants AI59694, LM009012, LM010098, EY022300, LM011360 and GM103534].
